# Transformational Leadership and Psychological Well-Being of Service-Oriented Staff: Hybrid Data Synthesis Technique

**DOI:** 10.3390/ijerph19138189

**Published:** 2022-07-04

**Authors:** Hyun-Duck Kim, Angelita Bautista Cruz

**Affiliations:** 1 Department of Sport Marketing, Keimyung University, Daegu 42601, Korea; kimgolf@kmu.ac.kr; 2 Department of Physical Education, Keimyung University, Daegu 42601, Korea

**Keywords:** transformational leadership, mixed-method research, service industry employees, nurse, Leximancer, systematic meta-analysis, psychological health

## Abstract

Leaders play a significant role in organizations and their leadership behaviors can either enhance or undermine the well-being of their employees. This study aimed to meta-analyze the relationship between transformational leadership and well-being in the service industry, and how employees’ gender and service sector moderated the strength of this relationship. This study used a convergent mixed-method approach. PubMed, MEDLINE, Google Scholar, AMED, and Scopus electronic databases were utilized to search for relevant studies. Textual data were analyzed using a text data-mining technique (Leximancer) to determine the relevant themes and concepts. Statistical data were examined through a comprehensive meta-analysis to determine their effect sizes. The qualitative results outline the major themes that emerged: leadership, well-being, and health. The quantitative findings revealed that the perceived well-being of male employees and those working outside of the health-care service sector was positively higher when employees’ leaders showed transformational leadership. In general, the findings from the qualitative and quantitative data converge. The findings confirm the positive relationship between transformational leadership and employee well-being. This study also highlights the applicability of a convergent mixed-method approach as a useful methodological strategy when analyzing both lexical and statistical data.

## 1. Introduction

People working in various service industries provide their clients or customers intangible services. For instance, customer service employees in the banking sector assist customers in managing, saving, and investing their money. Chefs cook high-quality and delicious meals and waiters courteously serve the dishes to customers. Healthcare workers provide assistance to patients and/or people with medical conditions and respond quickly during emergency situations [1,2,3,4]. These occupations generally require a great deal of interaction with clients, customers, or patients in order to satisfy the requirements of the services. In addition, as a result of the changing landscape of service work, wherein consumers are more demanding in terms of the quantity and quality of services, the sector is characterized by labor shortages, high turnover rates, organizational changes focused on technology, fast service, and multi-tasking. Thus, current and new employees are compelled to adapt to and cope with additional work loads. Fulfilling the required duties and responsibilities, while maintaining first-rate worker–customer interactions, are work demands that place great stress on workers. The stressful work demands often experienced by workers can potentially lead to unpleasant emotional and psychological reactions if the stressors persist [5,6,7]. If the stressors are not properly recognized and regulated, undesirable outcomes regarding workers’ well-being may occur. It is therefore important that organizations provide a conducive working environment for their workers by identifying the various work demands that can cause severe stress on service-oriented workers, and immediately reduce these stressors to safeguard workers’ well-being.

Employee well-being is described in many ways and comprises both positive and negative definitions. More positively, it is defined as employees’ subjective experiences of pleasure and positive feelings of vitality [8,9], such as contentment, satisfaction, serenity, personal growth, and learning vigor [9]. In contrast, it is negatively defined as the subjective experiences of negative affects such as stress, frustration, and anxiety [10]. In terms of well-being in the workplace, employee well-being can be conceptualized as job satisfaction, morale, job-related stress or tension, job-related depression, and job-related burnout [11,12]. Hence, the level of perceived satisfaction, stress, depression, or burnout of employees in various service-oriented occupations is dependent on the degree or frequency of positive and/or negative experiences encountered while in the workplace. These experiences may be work load and/or interactions with customers, co-workers, and superiors. The manner in which employees respond to these experiences determines their well-being.

Several factors have been shown to affect the well-being of occupational workers in various service-oriented industries. These factors include age [1], career goals [6], organizational support [5,6,7], workplace environment [2,5,13,14], and leadership behavior [1,7,15,16,17].

Studies about leader behaviors in the workplace have shown various influence on service-oriented staff’s psychological state. For instance, leadership behaviors that disregard employees’ concerns, avoid responsibility and accountability, and ignore open communication are reported to negatively affect bank employees’ mental health [14]. Similarly, in a study of call center agents, supervisory support and autonomy were negatively correlated with exhaustion [18]. Nurses reported a lower perceived overall mental health status when their leaders did not provide guidance, lacked involvement, and provided few work-related instructions [19]. In contrast, human resources employees had higher perceived well-being when their leaders encouraged creative thinking when solving problems, communicated goals and visions clearly, and had positive leader–follower relationships [7,16]. Healthcare workers caring for COVID-19 patients conveyed higher levels of perceived psychological well-being when their leaders emphasized the value of safety in the workplace and provided clear information about safety-related events and injury risks associated with the occupation [20]. Similarly, faculty members and academic staff teaching and working in higher educational institutions reported better quality of life when they thought their leaders provided them with motivation, intellectual stimulation, and individual consideration, and when leaders displayed idealized leadership attributes and behaviors. The above findings show that leadership behaviors (that is, the behavior of managers or supervisors) can negatively or positively affect the well-being of employees. This suggests a relationship exists between leaders’ leadership styles and/or behaviors and employees’ well-being. Further, this underscores positive leadership as a significant element in employees’ work-related outcomes.

### Theoretical Background and Literature Review

Transformational leadership is a well-known theoretical approach in explaining the importance of positive leadership for followers’ affect, cognition, and behaviors. According to transformational leadership [21], transformational leaders influence their followers to accomplish tasks and/or goals by providing inspiration, challenges, empowerment, and consideration to their followers. This positively changes followers’ trust, values, and perceptions, leading to higher levels of performance and motivation. Transformational leadership has four components: (1) idealized influence occurs when transformational leaders try to gain followers’ respect and trust by being a role model and following high ethical standards in the workplace; (2) inspirational motivation relates to the frequency with which transformational leaders motivate followers by providing values and a vision about the goals to be accomplished; (3) intellectual stimulation relates to the frequency with which transformational leaders encourage followers to think outside the box, and enhance their creativity and autonomy; and (4) individualized consideration relates to the frequency with which transformational leaders show their concern and support for followers’ needs by genuinely listening to followers’ aspirations and difficulties within and even outside the workplace. Given that transformational leaders demonstrate positive behaviors that stimulate intellectual capacity, cultivate relationships, and encourage competence and skills development, followers’ attitudes toward their leader and their work, in addition to their personal motivation in executing their assigned tasks, tend to be positively affected. Further, followers’ positive perceptions of work-related outcomes can lead to better well-being. In contrast, if leaders’ behaviors do not exemplify transformational leadership and followers perceive this, it can result in negative attitudes toward the leader and work environment, and potentially lead to a decline in followers’ well-being.

Given the vast scope of the published empirical studies related to transformational leadership and employee outcomes [11,16,17,18,19,20], several researchers have attempted to summarize previous findings using different approaches. These attempts aimed to further understand the impact of transformational leadership on employees’ work-related well-being. For instance, one study [4] conducted a systematic review of studies on the impact of leaders and their leadership behaviors on employee stress and affective well-being. It was found that transformational leadership was associated with lower stress levels and positive affective well-being in employees. Notably, the authors conducted their review process by identifying, screening, extracting, tabulating, synthesizing, and analyzing the relevant textual information on the topic. This type of review is generally more time consuming and prone to selection or attrition bias, and the reporting of findings may vary [22]. Hence, a methodological approach that can more efficiently and effectively code and synthesize the vast amounts of information and data from published studies may provide new insights. It may also reduce or even eliminate researcher bias, offer deeper interpretations, or present new themes about the relevant predictor and outcome variables relevant to this topic.

Other authors focused on summarizing the relevant previous studies using quantitative statistical data and analyzed them via meta-analysis [23,24]. Montano et al. [23] found negative associations between transformational leadership and burnout, stress, and affective symptoms. They also found a positive association between transformational leadership and positive well-being. Teetzen et al. [24] found significant positive associations between transformational leadership and employees’ affective–motivational well-being and pleased–relaxed well-being. They also reported that transformational leadership was negatively associated with the depressed–exhaustive and irritated–distressed well-being of employees. The findings of these meta-analyses confirmed not only the importance of leadership in developing employees’ well-being, but also the manner in which work-related factors may mediate this relationship. However, these meta-analyses did not consider other situational or personal variables that may moderate the effects of transformational leadership and employee well-being. These considerations are important when determining for whom, and in which situations, the relationship between transformational leadership and well-being is deemed pertinent. For instance, a significant difference between the genders was found in the relationship between transformational leadership and career satisfaction [25], suggesting that employees’ personal characteristics are an important factor that may influence the transformational leadership and well-being relationship.

Regarding situational variables, previous studies investigating the association between transformational leadership and well-being have not explored the possibility that the service industry of the employees may be a moderating factor. According to Sivanathan et al. [26], organizational and/or occupational identity are potential variables that can affect the relationship between transformational leadership and well-being. They proposed that leaders demonstrating transformational leadership can affect individuals’ sense of belonging or level of identification with the organization or occupation. Organizational identity is described as an employee’s level of perceived cohesion with their organization, whereas occupational identity is an individual’s sense of cohesion with their occupational group [26]. Although employees’ well-being may be positively affected by transformational leaders, this relationship may depend on the service sector in which the employees work, since employees’ degree of identification with their occupation or organization can be impacted by leadership behaviors through leaders’ interaction level with their employees [27].

Therefore, the employees’ gender and service sector are variables worthy of investigation to further understand how work-related variables moderate the relationship between transformational leadership and well-being. However, these two relevant variables have not been examined in previous meta-analysis studies. Understanding how the impact of transformational leadership on employees’ well-being may differ between male and female employees, and between the healthcare and non-healthcare service sectors, may extend our theoretical knowledge regarding transformational leadership and its consequences. In particular, it may help in identifying the moderating variables that may change the strength of the relationship between transformational leadership and work-related outcomes. Further, the findings may assist leaders, managers, and occupational psychologists in creating intervention programs to enhance leaders’ transformational leadership skills, which, in turn, would facilitate the enhancement of employees’ well-being. The mixed-method approach utilized in this study, specifically, the convergent parallel mixed-method approach [28], can help in examining both statistical and textual data, thereby providing more robust and in-depth inferences about the relevant variables. Thus, this study offers a novel research procedure for understanding transformational leadership and well-being by comparing textual and statistical data derived from qualitative and quantitative research method databases.

Hence, the current study aimed to systematically review and meta-analyze studies focusing on the relationship between transformational leadership and well-being in the service industry using a mixed-method approach. To address the research questions, a combination of data-mining and meta-analysis were adopted, to increase the breadth and depth of our understanding of the data, as suggested by Schoonenboom and Johnson [29]. The following research questions were explored in this study: (1) What major themes and concepts would emerge from synthesized textual data on transformational leadership and well-being? (2) What is the overall effect size (ES) of the relationship between transformational leadership and well-being? (3) Does gender moderate the relationship between transformational leadership and well-being? (4) Does the service sector of employees moderate the relationship between transformational leadership and well-being? (5) Do the findings from qualitative and quantitative studies show convergence or divergence?

## 2. Materials and Methods

Systematic reviews and meta-analyses have been adopted by an increasing number of scholars in the field of healthcare. This indicates that most systematic reviews and meta-analyses have been retrospective, without thoughtful considerations of empirical evidence from previous studies [30]. The methodological procedure used in this study is both qualitative and quantitative; it involves selecting, analyzing, and synthesizing data (that is, text data and numerical statistic results) to make novel inferences from the data-mining and meta-analysis findings. This methodological procedure is divided into two phases in terms of synthesizing data. The authors term the Hybrid Data Synthesis Technique (HDST).

### 2.1. Phase 1. Systematic Review and Data Mining

#### 2.1.1. Phase 1 Text Data Source Selection

**Figure 1:**
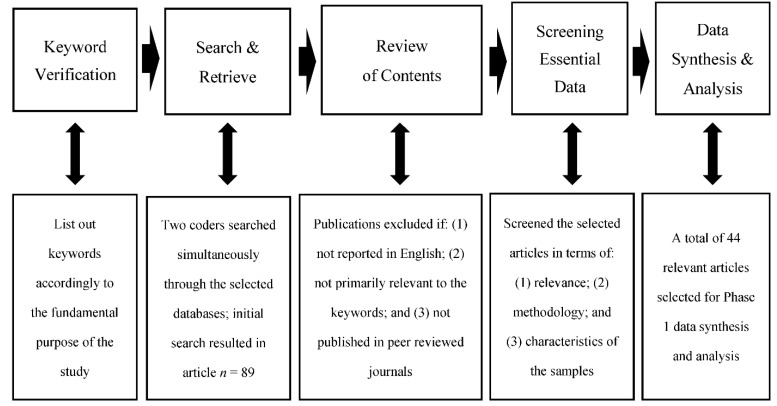
Procedural details of text data coding and analysis.

Data sources for Phase 1 analysis were selected using the following databases: PubMed, MEDLINE, Google Scholar, Allied and Complementary Medicine Database (AMED), and Scopus. The two authors of the study conducted the initial search strategy from the given databases concurrently using a range of combined related keywords, such as “psychological well-being”, “psychological state”, “healthcare”, “service industry”, “transformational”, “leadership”, “leadership behaviors”, “transformational leader”, “staff”, and “employee”. In addition to the keywords, the year of publication was set from 2000 to 2021 to search for potential articles. Studies were included if they were: (1) written in the English language; (2) had both psychological well-being and transformational leadership as focal study variables; and (3) were published in peer-reviewed journals. From the initial literature search, 89 published articles were identified. Reference lists of the preliminary searched articles were also checked for articles that could be relevant to the present study. Then, the authors independently searched and retrieved relevant documentation by screening content according to the inclusion criteria. Documents were carefully reviewed and excluded if (1) the document contained only a fractional amount of information relevant to this study’s purpose; (2) an article was a duplicate of a graduate thesis or dissertation; (3) the full text of each article was inaccessible; (4) sample participants (followers) were not within the service industry; and (5) there was a disagreement regarding the selection of the article because of its relevance. In terms of (5), the authors thoroughly discussed whether an article satisfied the selection criteria but were not able to achieve 80% consensus. Per the selection criteria, a total of 44 relevant articles conducted using both qualitative and quantitative methods were selected for Phase 1 of the analysis. The included articles were transformed into an analyzable format for further data synthesis and analysis (see Figure 1).

#### 2.1.2. Data-Mining Analysis

The initially collected text data of the 44 articles were examined utilizing a text data-mining technique with the Leximancer software, version 5.0 (Leximancer Pty Ltd., Brisbane, QLD, Australia). This software supports the visualization of text data by verifying and presenting thematic and conceptual maps; that is, it is able to reveal or highlight how themes and concepts are connected or grouped together. One of the significant advantages of this data mining technique is its ability to visualize focal text data topics. Visualizing the text data helps to understand the text data’s inherent structure [31]. Leximancer has been widely accepted as a text analytical tool that automatically generates a thesaurus of words and phrases based on contextual similarities, and semantic and relational extraction [32]. The visual outcome resulting from the concept and theme-mapping algorithm of this analytic tool is based on the theoretical framework of Bayesian decision theory [33,34]. Leximancer’s concept map is able to present the relative co-occurrence of words and phrases as equivalent thematic and conceptual networks [32]. After analyzing the data, Leximancer can deliver bubble-shaped clusters of “concepts” transformed or grouped as “themes” within relative keywords [31,32]. To understand Leximancer’s concept map, the following features are important: colors of the clusters represent the level of importance of each theme (that is, ranging from warm colors such as red and orange (higher connectedness to the theme), to cool colors such as blue and green (lower connectedness to the theme)); and size of a concept’s dot reflects its connectedness within the concept map of the program (for more information, refer to the Leximancer user guide, release 5.0).

### 2.2. Phase 2. Meta Analytic Review

#### Phase 2 Coding Data for Meta-Analytic Review

In Phase 2 of this study, a meta-analysis of the selected articles was carried out to extensively explore the text data retrieved from Phase 1. Meta-analysis has been widely adopted by scholars from a variety of academic fields to combine findings obtained from studies on equivalent subjects or domains [33,35]. This analytic method is known as the analysis of analyses, and is able to generate a general ES and confidence interval for the cumulative evidence of two or more studies [36,37,38]. The focal statistical index from a meta-analysis is the ES, which represents the degree of relation among study variables within the analysis [37,39].

**Figure 2:**
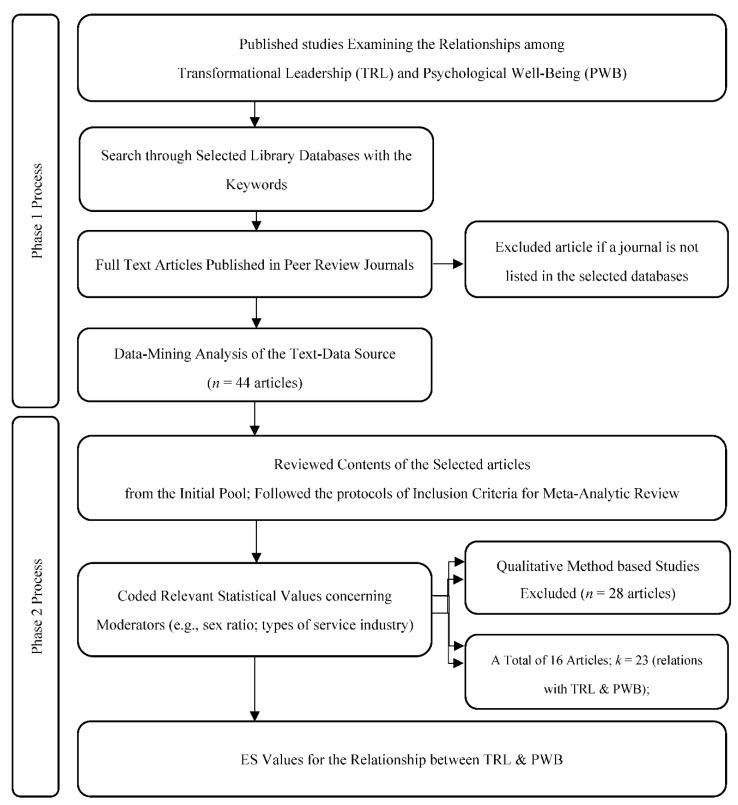
Flow diagram of the Hybrid Data Synthesis Technique for the Phase 1 and 2 analyses. Note: TRL = transformational leadership; PWB = psychological well-being; *n* = total sample size; *k* = total number of relations (correlation coefficient values) among independent variables and dependent variables.

**Figure 3:**
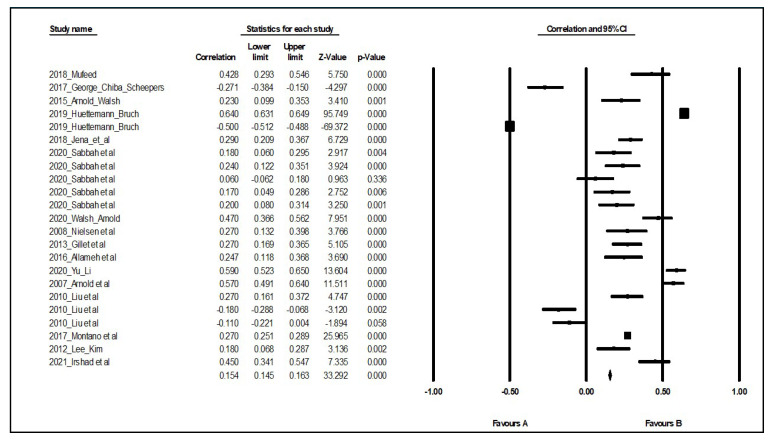
Forest plot of studies included in the quantitative analysis [11,16,20,23,47,48,49,50,51,52,53,54,55,56,57].

For the Phase 2 analysis, we followed the essential protocols suggested by the Preferred Reporting Items for Meta-Analysis (PRISMA) guidelines [40]. From the initial pool of 44 articles, studies that used qualitative design were excluded. A total of 16 studies (having a total of 28,687 samples) qualified for meta-analysis under Phase 2 (see Figure 2 and Supplementary File S1). Importantly, the selected articles were sorted into different moderators, namely, (1) gender ratio (i.e., proportion of gender for the target population) and service sector (healthcare vs. non-healthcare industry). The procedural details of Phases 1 and 2 are presented in Figure 2. Prior to the actual meta-analysis, heterogeneity was examined using the funnel plot. Results showed that the effect estimates extracted from each study were mostly positioned in the top area, which indicates that the studies had large sample sizes. Moreover, the standard error and Fisher’s Z values were above 0.10 and 0, respectively (see Supplementary File S2). Similarly, the forest plot showed variations in results among studies (Figure 3). These findings verified the assumption of heterogeneity and, therefore, a random-effects model was selected to analyze relevant data [41,42]. Procedures and formulas for conducting random effects meta-analysis were applied following the recommendations of Hunter and Schmidt [43] and other authors who conducted meta-analytic studies related to leadership [44,45]. Relevant statistical data, such as correlations and sample sizes obtained from the quantitative studies, were computed using the comprehensive meta-analysis software (CMA) to estimate ES values and 95% credibility intervals [36]. An ES greater than or equal to 0.4 is interpreted as large, 0.25–3.99 as moderate, and less than 0.25 as small [46].

## 3. Results

### 3.1. Results of the Data-Mining Analysis

**Figure 4:**
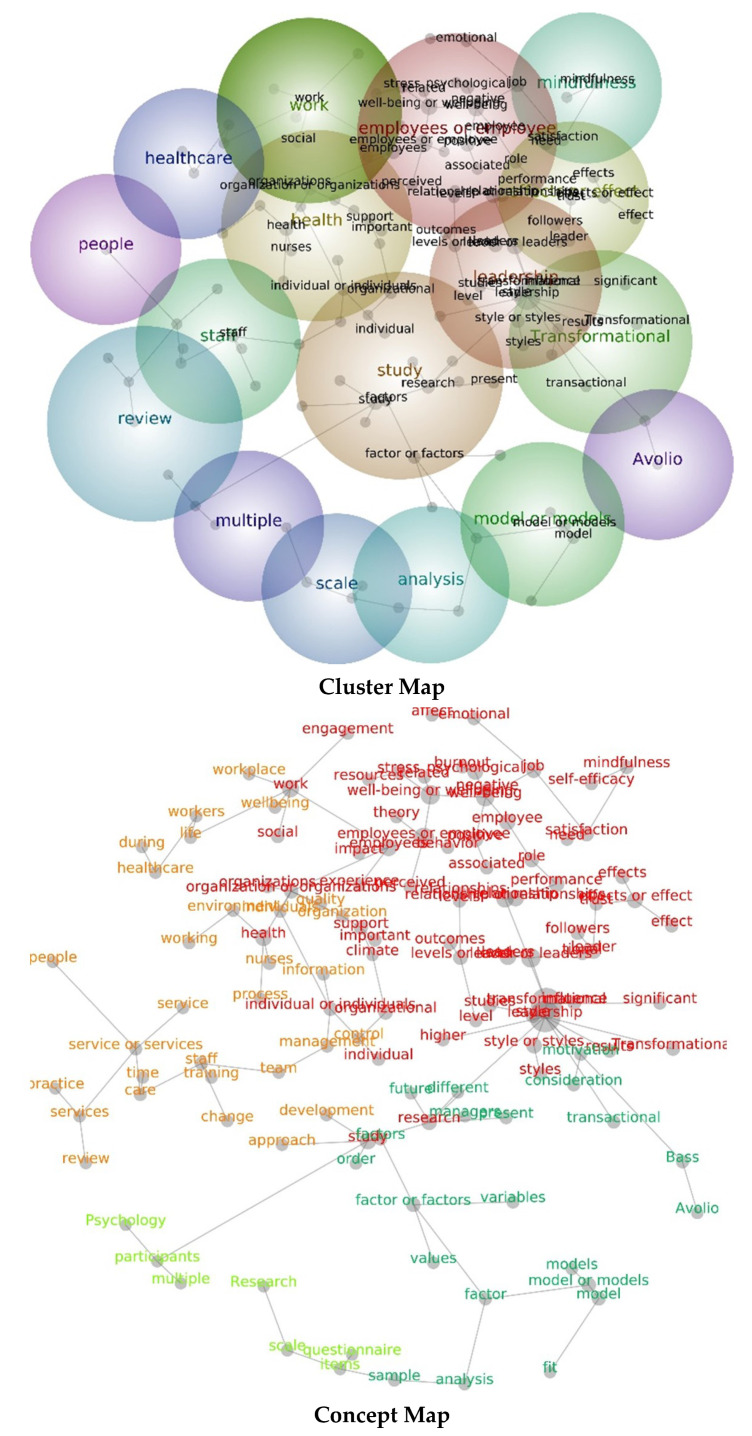
Theme and concept map for the selected articles on transformational leadership and psychological well-being. The relative size of each theme circle is the boundary for clustered concepts; warm colors (red, orange) indicate the most important themes and cool colors (blue, green) denote less important themes.

As described in the methodology section, this study’s analyses were performed in two phases. We first analyzed the selected articles on transformational leadership and psychological well-being utilizing data-mining technology. This procedure verified that the themes and concepts merged in the text data of the selected articles; that is, the common themes and concepts across the selected articles were revealed through a semantic extraction process. The common themes and concepts are presented in Figure 4.

Leximancer data-mining software provides a graphical representation (also called a “concept/cluster map”) of the themes and concepts of a text data source. The concept map shows differences in the size and color around “words”. The colors and dot sizes indicate the relevance of a theme or concept, and the degree of mathematical connectedness among words [32]. The data-mining analysis conducted in Phase 1 verified a total of 13 significant themes across the selected articles, among which “leadership” had the strongest significance in terms of hit count and connectedness, followed by “well-being”, “health”, “work”, “effect/effects”, and “study.”

The thematic and conceptual structures of the selected articles are presented in Table 1.

### 3.2. Results of the Meta-Analysis

After confirming the significant themes and conceptual structures in the selected articles, the authors carried out a systematic review of the articles through a meta-analysis. The procedural details of the data coding for this meta-analysis are thoroughly portrayed in Figure 2. In Phase 2 of the Hybrid Data Synthesis Technique, among the 16 included studies, 13 were cross sectional, one was longitudinal, one was time-lagged, and one was a meta-analysis. Participants’ perceptions of their leaders’ transformational leadership were measured via the actual or adopted version of the Multifactor Leadership Questionnaire (seven studies) [58], Global Transformational Leadership Scale (four studies) [59], and Transformational Leadership Behavior Inventory (TLI) (two studies) [60]. Two studies used questionnaires developed by other authors to measure transformational leadership [61,62]. Well-being as an outcome measure was defined and evaluated differently among the studies. These well-being outcomes were job-related well-being (e.g., quality of work life), job stress, emotional exhaustion, positive emotions, physical and mental well-being, engagement, and satisfaction. Participants in the healthcare sector were employees of hospitals, health centers, and elderly care facilities (seven studies), whereas other service-oriented employees worked in education, customer service, banking, insurance, retail, and IT institutions or companies (nine studies).

Results of the meta-analysis showed moderate to large effects in the relationship between transformational leadership behavior and psychological well-being across gender groups and service sectors. The ES of transformational leadership on well-being was lower in female employees (ES = 0.297; 95% CIs = 0.184, 0.401; *k* = 12; *p* < 0.05) than males (ES = 0.382; 95% CIs = 0.158, 0.568; *k* = 7; *p* < 0.05). With regard to the service sector, the ES of transformational leadership on well-being was lower in healthcare employees (ES = 0.265; 95% CIs = 0.169, 0.355; *k* = 11; *p* < 0.05) than in non-healthcare service employees (ES = 0.411; 95% CIs = 0.211, 0.578; *k* = 8; *p* < 0.05); see Table 2.

## 4. Discussion

### 4.1. Data-Mining from Qualitative Studies

The current study examined the relationship between transformational leadership and employee well-being, and how personal (employee gender) and situational (type of service sector) factors moderated the predictor and outcome variables using a mixed-method approach. Based on the qualitative data processed using Leximancer, the results showed three major themes emerged from the relationship between transformational leadership and well-being, namely, leadership, well-being, and health. The leadership theme comprised concepts such as transformational, leader, styles, role, followers, support, influence, and outcomes. These word concepts connected to the leadership theme suggest a general idea of how leadership is depicted; that is, the leaders’ styles of influencing or supporting followers to achieve outcomes [63]. The second major theme is well-being, to which pertinent concepts such as relationships, employee, positive, organizational, effects, satisfaction, stress, mindfulness, negative, trust, emotional, performance, job, and engagement were strongly connected. Based on the interconnectivity of concepts, this theme denotes how well-being is examined in an organization and conceptualized as an outcome variable within the transformational leadership literature; that is, well-being is related to either a positive (satisfaction, engagement) or negative (stress) psychological state experienced by employees in the workplace. The third major theme that emerged is health. The relevant word concepts connected with this theme are employees, study, factors, management, environment, future, life, care, and nurses, which indicate the work setting (healthcare) and participants (nurses) that were investigated in the included studies.

Other minor themes also emerged but with fewer concepts linking them with other themes, such as Avolio, model, analysis, scale, review, and staff. The conception of Avolio as a theme, which has a connection with the word concept Bass, is worth noting. This result suggests that the names of the authors who created the transformational leadership theory, and the value and impact of these authors in the field of organizational and positive psychology, in general, and in the area of leadership in particular, are highlighted in the included studies.

Overall, based on the lexical data from the consolidated qualitative and quantitative studies, the interconnection of the emergent themes and concepts related to transformational leadership and well-being suggests that these variables are strongly related with one another, and underscores how leadership can either develop or undermine the well-being of service-oriented employees.

### 4.2. Meta-Analysis from Quantitative Studies

The findings showed that the impact of transformational leadership has a positive and moderate effect on employees’ well-being. This suggests that, when leaders in the service industry exhibit idealized influence, promote self-efficacy in employees, challenge employees to think for themselves, and build sincere relationships with employees, they tend to promote employees’ positive well-being. In turn, this highlights the importance of transformational leadership in developing greater levels of well-being, and even work effectiveness, in employees. This finding is consistent with previous studies [23,26,27].

Interestingly, the current study found that the effect of transformational leadership on employee well-being was higher in male employees than in their female counterparts. This finding suggests that perceptions of well-being tend to be higher in male employees compared with female employees when leaders demonstrate high levels of transformational leadership behaviors. This result contradicts previous findings that showed that transformational leadership has a bigger impact on women’s work satisfaction than that of men [25]. This disparity may have arisenbecause the study participants from previous research were mainly engineering and/or computing employees, whereas the samples of the present study comprised of employees working in the service industry-related sectors. Furthermore, the current result corroborates the notion that, when leader and follower leadership perceptions are highly congruent, it can positively influence followers’ work outcomes [64,65,66]. In the present study, the higher level of perceived positive well-being of male employees compared to female employees may have resulted from male employees’ perceptions about effective transformational leadership behaviors demonstrated by their leaders. A previous study showed that male and female employees had different views about effective leadership [66]. In particular, more male than female employees described effective leadership as exemplifying fairness, equality, and honesty. Similarly, when workers were asked how leadership could be implemented in order to facilitate employee effectiveness in the workplace, more male than female employees reported that leaders should emphasize equality, fairness, and honesty in their followers. In contrast, more female than male employees stated that leaders should offer support and developmental opportunities, and provide contingent rewards, in order to enhance their work efficiency. In this case, male employees’ perceptions of effective leadership in terms of fairness, equality, and honesty corresponded to the idealized influence element of transformational leadership. In contrast, female employees’ perceptions of effective leadership, as characterized by developmental and supportive behaviors, aligned with the individualized consideration element of transformational leadership and transactional leadership behaviors. Following this notion, male employees’ well-being tended to be positively affected to a greater degree than female employees; this is because the transformational leadership behaviors that male employees perceived as being effective and facilitating their work efficiency—in this case, idealized behavior—were highly aligned with how the leader valued and personified this leadership behavior (i.e., placing high value on commitment, honor, and integrity) to their followers [67]. Conversely, the perceived positive well-being of female employees was slightly lower because the transformational leadership behaviors they recognized as important were less exemplified by their leader. Given this finding, this study provides additional support to the leadership literature on transformational leadership as an effective form of leadership for enhancing the well-being of employees, in general, and those in the service industry, in particular. Furthermore, the current study also extends our knowledge in relation to leadership by showing that employees’ gender is a personal moderating factor in the relationship between transformational leadership and well-being.

Based on the results of the meta-analysis, the effect of transformational leadership on the well-being of employees differed between service industry sectors; that is, the well-being of non-healthcare employees was more significantly enhanced than that of employees working in the healthcare service sector when leaders displayed transformational leadership. The higher perception of well-being among employees outside the healthcare sector may be attributable to employees’ level of interaction with their leaders [27]. According to social exchange theory [68], the interaction between two entities involves a cost–benefit analysis, such that when the benefits of the interaction are greater than the cost, the interaction may commence or continue. Following this assumption, transformational leaders may have engaged in higher-quality interactions and demonstrated more frequent transformational leadership behaviors to non-healthcare employees (e.g., bank employees, teachers). As a result of these positive leadership behaviors, employees develop trust, respect, and positive attitudes, not only toward the leader, but also to the organization or occupation [24], consequently affecting employees’ well-being in the workplace. Conversely, given that the main duties of healthcare workers involve taking care of patients, interacting with the leader may be perceived as being less important than connecting with patients. Hence, healthcare employees’ positive well-being was relatively lower, despite their leaders demonstrating transformational leadership. This finding offers further understanding of the effects of transformational leadership on employees’ well-being, and underscores that the employees’ service sector significantly moderates this relationship.

### 4.3. Convergence between Themes/Concepts and Statistical Results

Comparing the findings from both qualitative and quantitative studies related to transformational leadership and well-being, leadership as a theme, and the related concepts that emerged from the qualitative studies, provided support for the definition and conceptualization of transformational leadership in quantitative studies. The well-being theme and the relevant concepts connected to it denote the importance of well-being in the workplace, and the manner in which well-being is conceptualized and assessed as an outcome variable in the quantitative studies that were examined. Interestingly, the concepts of trust and mindfulness included in the well-being theme are somewhat inconsistent as outcome variables, as defined and measured in quantitative studies. It is unclear why these two concepts are strongly connected with the well-being theme; however, trust (in one’s management or leader) has been shown to be associated with transformational leadership [69,70], whereas mindfulness has been found to be negatively correlated with perceived stress, depression, and anxiety, which are associated with psychological well-being [71]. Hence, although these two concepts may not be directly congruent with the manner in which well-being is characterized in quantitative studies, trust appears to have an indirect link with well-being given its connection with transformational leadership, and therefore serves as a potential psychological mechanism that connects transformational leadership and well-being [26]. By comparison, mindfulness may be regarded as a coping strategy to maintain or enhance psychological well-being, or as a safeguarding strategy against stress [71].

An interesting finding is the appearance of the word “nurse” as a relevant concept included within the health theme. This concept was also interconnected with the healthcare and work themes, likely reflecting the sample participants that were classified under the healthcare sector, who were mostly nurses. This finding highlights the manner in which researchers generally personify healthcare professionals as nurses. Another noteworthy result from the qualitative analysis is that no words related to gender (e.g., female, male) emerged as a relevant concept pertaining to transformational leadership and well-being. This surprising result seemed to reinforce the importance of examining this variable in our meta-analysis. This demonstrates how qualitative reports, which are mostly excluded from many systematic reviews and meta-analyses, may provide valuable information that can help researchers identify other factors that may be interesting to explore quantitatively, so that we can further understand a particular research topic.

### 4.4. Practical Implications

Based on the findings of the current study, it is imperative that leaders who display transformational leadership (idealized influence, inspirational motivation, intellectual stimulation, and individualized consideration) are mindful about their employees’ gender and workplace setting. Specifically, they should be mindful of how female employees and healthcare employees perceive effective leadership. Identifying the transformational leadership behaviors recognized by employees as being crucial for the enhancement of their well-being can assist leaders in terms of the transformational leadership behaviors they need to regulate (either by reducing or increasing the behavior’s frequency). Subsequently, when leaders demonstrate the appropriate behaviors that are congruent with the effective leadership behaviors identified by followers, followers’ well-being is likely to be enhanced. For instance, if female employees, in order to be happy and productive at work, acknowledge their independence and value their privacy, leaders should ensure provision of a higher degree of intellectual stimulation but slightly restrain showing individualized consideration. Similarly, when healthcare employees, particularly nurses, are assigned to a high-risk area in the hospital, such as intensive care or a highly communicable disease control unit, leaders should exhibit frequent individualized consideration behaviors to promote positive relationships with followers. Such actions would help reduce the employees’ stress and anxiety during work, and thereby enhance their well-being.

### 4.5. Limitations

This investigation only analyzed the direct effect of transformational leadership on employees’ well-being based on previous studies that computed overall transformational leadership scores. In this regard, quantifying the individual effect of each dimension of transformational leadership on employees’ well-being is not yet viable due to the insufficient availability of empirical studies. Future studies identifying the relative effects of each dimension of transformational leadership on employees’ well-being and other work outcomes are warranted in order to for us to further understand which transformational leadership dimensions enhance or undermine work-related outcomes in employees.

Since only studies published in scientific journals were included, publication bias is anticipated to a certain extent [72,73]. This point may be noticed in the asymmetric result of the funnel plot. However, as previously noted, this asymmetric result is likely due to the presence of heterogeneity rather than publication bias [62]. This notion was further verified by the inconsistency (*I*^2^) values above 75% which indicate high degrees of statistical heterogeneity [73]. Nonetheless, when conducting meta-analysis studies, it is suggested to utilize all possible resources when searching for articles (both published and unpublished), and when examining and interpreting findings, in order to avoid publication bias.

Finally, only employees’ gender and service sector were examined as moderating variables. It would be interesting to examine other leader-, follower-, or organization-related factors, such as leaders’ gender and experience, employee personality profiles, and other industries (e.g., sport, manufacturing), in order to gain a more comprehensive understanding of the impact of transformational leadership on followers’ outcomes.

## 5. Conclusions

This study made several important theoretical and practical contributions. First, transformational leadership has moderate to large effects on employee well-being. Second, the influence of transformational leadership can be moderated by employees’ gender and service sector. Third, the application of a mixed-model approach, which we termed the Hybrid Data Synthesis Technique (HDST), is a novel and promising procedure that can provide a more robust and comprehensive understanding of a research phenomenon, problem, or question by comparing the results drawn from both lexical and statistical data. More studies using this approach are encouraged for researchers who are examining both qualitative and quantitative studies.

## Figures and Tables

**Table 1:** Thematic and conceptual analysis results from the selected articles on transformational leadership and psychological well-being.

Theme	Hit Count	Connectivity	Concepts	Relevancy Rate (%)
Leadership	4171	105,907	Leadership	100
Well-being	2849	60,022	Transformational	100
Health	2081	37,305	Employee	40
Work	2673	32,403	Well-being	34
Effects/effect	1906	27,330	Leader/s	34
Study	1816	20,183	Work	31
Model/Models	762	12,343	Health	26
Nurses	467	11,794	Employees	25
Staff	268	11,394	Relationship/s	23
Sample	227	5165	Research	22
Transformational	416	4874	Job	20
Review	171	2655	Positive	19
Approach	141	1800	Performance	19
Avolio	125	748	Psychological effect	19
Practice	111	551	Model/s	18

Note. The relevancy rate is the percentage frequency of text segments coded with that particular concept from the analysis of each national origin.

**Table 2:** Meta-analysis on the relationship between transformational leadership behavior and employees’ psychological well-being for different genders and service sectors.

Moderator	Gender	*k*	ES	−95%CI	+95%CI	*Z*	*Q*	df(*Q*)	*I* ^2^
Gender Ratio	Overall	19	0.328	0.198	0.447	4.766	1720.919	18	98.954
	Female	12	0.297	0.184	0.401	5.035	132.091	11	91.672
	Male	7	0.382	0.158	0.568	3.253	1409.612	6	99.574
Service Sector	Healthcare	11	0.265	0.169	0.355	5.292	75.020	10	86.670
	Non-healthcare	8	0.411	0.211	0.578	3.852	1413.906	7	99.504

Note. *k* = number of correlations; *Q* = homogeneity statistic; CI = confidence interval; ES = weighted random effect size.

## Data Availability

Not applicable.

## Supplementary Materials

The following supporting information can be downloaded at: https://www.mdpi.com/article/10.3390/ijerph19138189/s1, Supplementary File S1: List of studies included in the quantitative analysis. Supplementary File S2: Funnel plot of standard error by Fishers’ Z. Supplementary File S3: List of qualitative studies [17,70,74,75,76,77,78,79,80,81,82,83,84,85,86,87,88,89,90,91,92,93,94,95,96,97,98,99].
