# Supplementary material for: Transformational Leadership and Psychological Well-Being of Service-Oriented Staff: Hybrid Data Synthesis Technique
Source: Int J Environ Res Public Health. 2022 Jul 4;19(13):8189. doi: 10.3390/ijerph19138189 (PMC9266046; doi:10.3390/ijerph19138189)
Supplement: Supplementary file 1 [file ijerph-19-08189-s001.zip › Supplementary File S1 Studies in meta.pdf]

Authors	Year	Sample Size	Outcome	Service Sector	Gender Proportion
Mufeed [47]	2018	161	Quality of work life	Education	Male high
George et al. [48]	2017	242	Job stress	Education	Male high
Arnold and Walsh [16]	2015	215	General health	Customer service	Male high
Huettermann and Bruch [49]	2019	15952	Emotional exhaustion, engagement	Customer service/management	Male high
Jena et al. [50]	2018	511	General well-being	Service employee	Male high
Sabbah et al. [19]	2020	260	Work Effort, effectiveness, satisfaction, physical and mental health	Healthcare	Female only
Walsh and Arnold [51]	2020	246	Psychological well-being	Retail/sales/banking	Male high
Nielsen et al. [52]	2008	188	Positive mind state	Healthcare	Female high
Gillet et al. [53]	2013	343	Quality of work life	Healthcare	Female high
Allameh et al. [54]	2016	217	Overall health	Healthcare	Female high
Ding et al. [55]	2020	406	Psychological well-being	Diverse	Female high
Arnold et al. [56]	2007	319	Affective well-being	Healthcare	Female high
Liu et al. [11]	2010	297	Work stress, stress symptoms, job satisfaction,	Diverse	Male high
Montano et al. [23]	2017	8798	Multiple outcomes	Diverse	Male high
Lee and Kim [57]	2012	300	General well-being	Healthcare	Female only
Irshad et al. [20]	2021	232	Psychological well-being	Healthcare	Female high

Supplementary Table S1. List of studies included in the quantitative analysis
