# Supplementary material for: Transformational Leadership and Psychological Well-Being of Service-Oriented Staff: Hybrid Data Synthesis Technique
Source: Int J Environ Res Public Health. 2022 Jul 4;19(13):8189. doi: 10.3390/ijerph19138189 (PMC9266046; doi:10.3390/ijerph19138189)
Supplement: Supplementary file 1 [file ijerph-19-08189-s001.zip › Supplementary File S3 Quali studies.pdf]

Qualitative studies included in the data mining analysis

17. E-Rubbab, U.; Farid, T.; Iqbal, S.; Saeed, I.; Irfan, S.; Akhtar, T. Impact of Supportive Leadership During Covid-19 on Nurses' Well-Being: The Mediating Role of Psychological Capital. *Front Psychol*. 2021, 12, 695091. https://doi.org/10.3389/fpsyg.2021.695091.

70. Kelloway, E.K.; Turner, N.; Barling, J.; Loughlin, C.A. Transformational Leadership and Employee Psychological Well-Being: The Mediating Role of Employee Trust in Leadership. *Work Stress* **2012**, *26*, 39–55. https://doi.org/10.1080/02678373.2012.660774.

74. Nanjundeswaraswamy, T.S.; Swamy, D.R. An Empirical Research on the Relationship between Quality of Work Life and Leadership Styles in SMEs. Bangladesh E-J. Sociol. 2015, 12, 41–52.

75. McCabe, M.P.; Beattie, E.; Karantzas, G.; Mellor, D.; Sanders, K.; Busija, L.; Goodenough, B.; Bennett, M.; von Treuer, K.; Byers, J. (2018). A Randomized Controlled Trial to Evaluate the Effectiveness of a Staff Training Program to Implement Consumer Directed Care on Resident Quality of Life in Residential Aged Care. BMC Geriatr. 2018,18, 1–8. https://doi.org/10.1186/s12877-018-0966-1

76. McCabe, M.P.; Beattie, E.; Karantzas, G.; Mellor, D.; Sanders, K.; Busija, L.; Goodenough, B.; Bennett, M.; von Treuer, K.; Byers, J. Consumer Directed Care in Residential Aged Care: An Evaluation of a Staff Training Program. Aging. Ment. Health. 2020, 24, 673–678. https://doi.org/10.1080/13607863.2019.1574711

77. Zbierowski, P. Happy Followers of Positive Leaders. The Effect of Positive Leadership on Well-Being of Followers. J. Posit. Manag. 2019, 10, 51–61. https://doi.org/10.12775/JPM.2019.016

78. Bhatt, H.C. Leadership Styles and Quality of Work Life in Small and Medium Scale Enterprises of Kumoun Region of Uttarakhand. J. Strategic Hum. Resour. Manag. 2018, 7, 23–32.

79. Alimo-Metcalfe, B.; Alban-Metcalfe, J. More (good) Leaders for the Public Sector. Int. J. Public Sect. Manag. 2006, 19, 293–315. https://doi.org/10.1108/09513550610669167

80. Vries, J.; Koster, R.; Stam, D. (2016). Safety Does Not Happen by Accident: Antecedents To A Safer Warehouse. Prod Oper Manag. 2016, 25, 1377–1390. https://doi.org/10.1111/poms.12546

81. Grau-Alberola, E.; Figueiredo-Ferraz, H.; López-Vflchez, J.J.; Gil-Monte, P.R. The Healthy Management: The Moderator Role of Transformational Leadership on Health Workers. An. de Psicol. 2022, 38, 128–138. https://doi.org/10.6018/analesps.471121

82. Ahmed, O.M.; Ishak, A.K.; Kamil, B.A.M. (2019). Transformational Leadership and Employee Psychological Wellbeing among Faculty Staff: The Mediating Role of Self-Efficacy. IJMAE. 2019, 6, 184–197.

83. Hultgren, U.; Palmer, S.; O'Riordan, S. Can Cognitive Behavioural Team Coaching Increase Well-being? Coaching Psychologist. 2013, 9, 100–110.

84. Jambawo, S. Transformational Leadership and Ethical Leadership: Their Significance in the Mental Healthcare System. Br. J. Nurs. 2018, 27, 998–1001. https://doi.org/10.12968/bjon.2018.27.17.998

85. Moffat, J.; Sass, B.; Mckenzie, K.; Bhui, K. Enhancing Pathways & Mental Healthcare for BME groups: Learning between the Ideological and Operational. Int Rev Psychiatry. 2009, 21, 450–459. https://doi.org/10.1080/09540260802202075

86. i Solà, G.J.; i Badia, J.G.; Delgado Hito, P.; Campo Osaba, M.A.; Del Val García, J.L. Self-perception of leadership styles and behaviour in primary health care. BMC Health Serv. Res. 2016, 16, 1–9. https://doi.org/10.1186/s12913-016-1819-2

87. Cluff, C.C.; Colker, J.J. (2021). The Role of Interpersonal Exchange in Transformational Leadership. Prof. Saf. 2021, 66, 20–21.

88. Kensbock, J.M.; Boehm, S.A. The Role of Transformational Leadership in the Mental Health and Job Performance of Employees with Disabilities. Int. J. Hum. Resour. Manag. 2016, 27, 1580–1609. https://doi.org/10.1080/09585192.2015.1079231

89. Choy-Brown, M.; Stanhope, V.; Wackstein, N.; Delany Cole, H. (2020). Do Social Workers Lead Differently? Examining Associations with Leadership Style and Organizational Factors. Hum. Serv. Organ. Manag. Leadersh. Gov. 2020, 44, 332–342. https://doi.org/10.1080/23303131.2020.1767744

90. Garman, A.N.; Davis-Lenane, D.; Corrigan, P.W. Factor Structure of the Transformational Leadership Model in Human Service Teams. J. Organ. Behav. 2003, 24, 803–812. https://doi.org/10.1002/job.201

91. Parker, P. A.; Sorensen, J. Emotional Intelligence and Leadership Skills among NHS Managers: An Empirical Investigation. International Journal of Clinical Leadership. 2008,16, 137–142.

92. Vella, S.A.; Oades, L.G.; Crowe, T.P. A Pilot Test of Transformational Leadership Training for Sports Coaches: Impact on the Developmental Experiences of Adolescent Int J Sports Sci Coach. 2013, 8, 513–530.

93. Hill, D.M.; Brown, G.; Lambert, T.L.; Mackintosh, K.; Knight, C.; Gorczynski, P. Factors Perceived to Affect the Wellbeing and Mental Health of Coaches and Practitioners Working within Elite Sport. Sport Exerc. Perform. Psychol. 2021, 10, 504–518. https://doi.org/10.1037/spy0000263

94. Carleton, E.L.; Barling, J.; Trivisonno, M. Leaders' Trait Mindfulness and Transformational Leadership: The Mediating Roles of Leaders' Positive Affect and Leadership Self-Efficacy. Can. J. Behav. Sci. 2018, 50, 185–194. https://doi.org/10.1037/cbs0000103

95. Rowold, J.; Borgmann, L.; Bormann, K. Which Leadership Constructs Are Important for Predicting Job Satisfaction, Affective Commitment, and Perceived Job Performance in Profit versus Nonprofit Organizations? Nonprofit Manag Leadersh. 2014, 25, 147–164. https://doi.org/10.1002/nml.21116

96. Bryant, P.; Butcher, J.T.; O'Connor, J. Improving School Leadership: The Connection of Transformational Leadership and Psychological Well-Being of the Followers. School Leadership Review. 2016, 11 Available at: https://scholarworks.sfasu.edu/slr/vol11/iss2/6

97. Chu, H.; Qiang, B.; Zhou, J.; Qiu, X.; Yang, X.; Qiao, Z.; Song, X.; Zhao, E.; Cao, D.; Yang, Y. The Impact of Transformational Leadership on Physicians' Performance in China: A Cross-Level Mediation Model. Front. Psychol. 2021, 12, 586475. http://lps3.doi.org.kims.kmu.ac.kr/10.3389/fpsyg.2021.586475

98. Decuypere, A,; Audenaert, M,; Decramer, A. When Mindfulness Interacts With Neuroticism to Enhance Transformational Leadership: The Role of Psychological Need Satisfaction. Front Psychol. 2018, 9, 2588. doi: 10.3389/fpsyg.2018.02588.

99. Tafvelin, S.; Nielsen, K.; von Thiele Schwarz, U.; Stenling, A. Leading Well is a Matter of Resources: Leader Vigour and Peer Support Augments the Relationship Between Transformational Leadership and Burnout. Work & Stress. 2019, 33, 156–172. https://doi.org/10.1080/02678373.2018.1513961
