# Supplementary figures and images for: Transformational Leadership and Psychological Well-Being of Service-Oriented Staff: Hybrid Data Synthesis Technique
Source: Int J Environ Res Public Health. 2022 Jul 4;19(13):8189. doi: 10.3390/ijerph19138189 (PMC9266046; doi:10.3390/ijerph19138189)

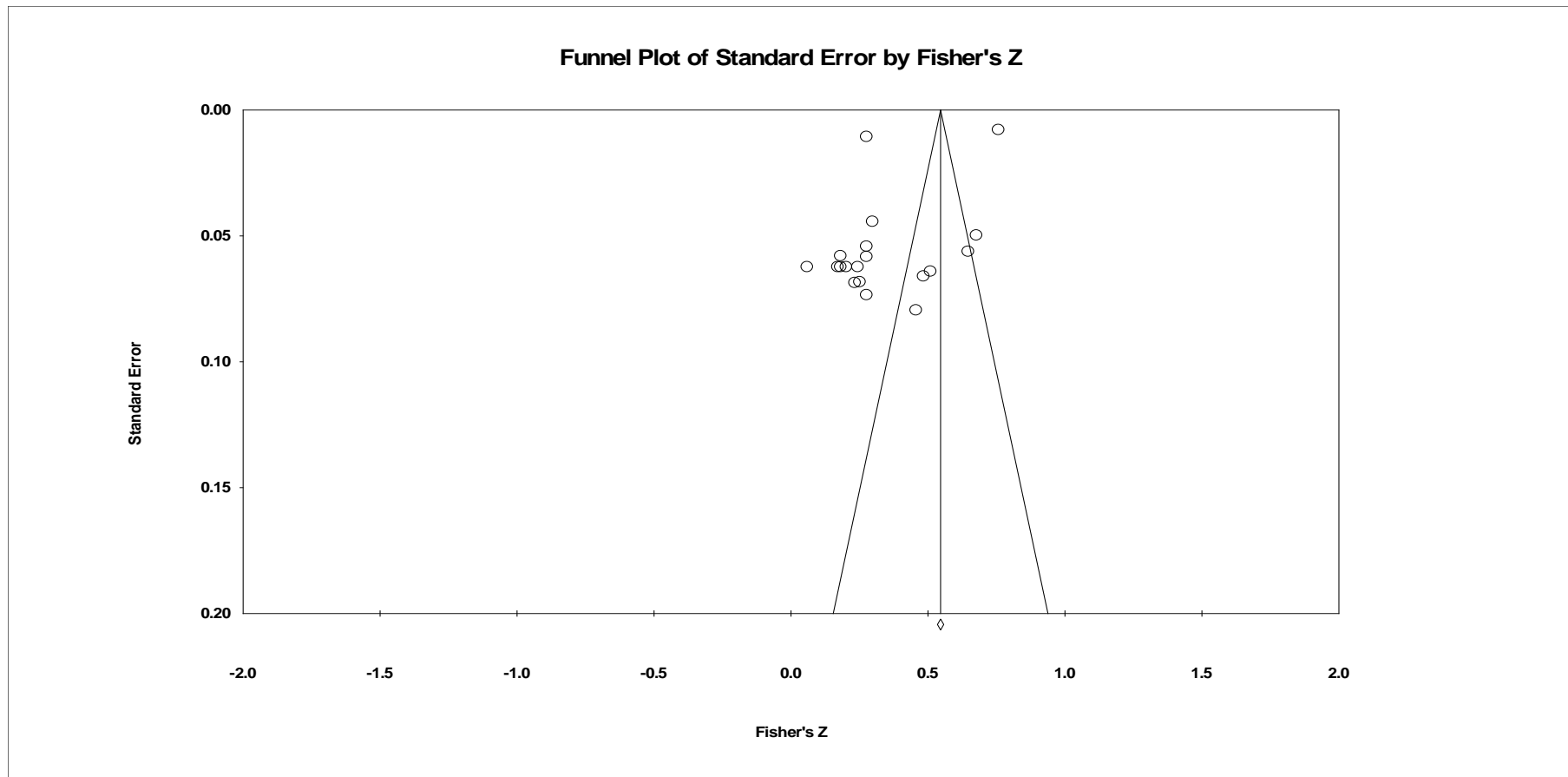

**Supplementary Figure S1.** Funnel plot of standard error by Fishers' Z

Supplement: Supplementary file 1 [file ijerph-19-08189-s001.zip › Supplementary File S2 Funnel Plot.pdf]
